# ﻿A new species of *Purpureocillium (Ophiocordycipitaceae)* fungus parasitizing trapdoor spiders in Brazil’s Atlantic Forest and its associated microbiome revealed through *in situ* “taxogenomics”

**DOI:** 10.3897/imafungus.16.168534

**Published:** 2025-12-12

**Authors:** João P. M. Araújo, Natalia A. S. Przelomska, Rhian J. Smith, Elisandro R. Drechsler-Santos, Genivaldo Alves-Silva, Kelmer Martins-Cunha, Tsuyoshi Hosoya, Janet J. Luangsa-ard, Allison Perrigo, Mar Repullés, Pável Matos-Maraví, Roseina Woods, Oscar A. Pérez-Escobar, Alexandre Antonelli

**Affiliations:** 1 Natural History Museum of Denmark, University of Copenhagen, Copenhagen, Denmark Royal Botanic Gardens Richmond United Kingdom; 2 Royal Botanic Gardens, Kew, Richmond, Surrey, UK University of Copenhagen Copenhagen Denmark; 3 School of the Environment and Life Sciences, University of Portsmouth, Portsmouth, UK University of Portsmouth Portsmouth United Kingdom; 4 MIND.Funga/MICOLAB, Botany Department, Santa Catarina Federal University, Florianopolis, Brazil Santa Catarina Federal University Florianopolis Brazil; 5 Department of Botany, Division of Fungi and Algae, National Museum of Nature and Science, Tokyo, Japan National Museum of Nature and Science Tokyo Japan; 6 National Center for Genetic Engineering and Biotechnology (BIOTEC), Thailand Science Park, Pathum Thani, Thailand National Center for Genetic Engineering and Biotechnology Pathum Thani Thailand; 7 Lund University Botanical Garden, Lund University, Lund, Sweden Lund University Lund Sweden; 8 Gothenburg Global Biodiversity Centre, Department of Biological and Environmental Sciences, University of Gothenburg, Gothenburg, Sweden University of Gothenburg Gothenburg Sweden; 9 Biology Centre of the Czech Academy of Sciences, Institute of Entomology, České Budějovice, Czech Republic Biology Centre of the Czech Academy of Sciences, Institute of Entomology České Budějovice Czech Republic; 10 Faculty of Science, University of South Bohemia, České Budějovice, Czech Republic University of South Bohemia České Budějovice Czech Republic; 11 Department of Biology, University of Oxford, Oxford, UK University of Oxford Oxford United Kingdom; 12 Antonelli Foundation for Biodiversity Research and Conservation, Nova Friburgo, Brazil Antonelli Foundation for Biodiversity Research and Conservation Nova Friburgo Brazil

**Keywords:** *
Ascomycota
*, entomopathogens, genomics, conservation, *
Ophiocordycipitaceae
*

## Abstract

Our planet is inhabited by an estimated 2.5 million species of fungi, of which fewer than 10% have been scientifically described. Some of the most understudied yet remarkable fungal species are those capable of parasitizing arthropods, notably insects and spiders. Here, we explore the hidden diversity of a spider-attacking (araneopathogenic) fungus and its associated microbiome in one of the world’s most biodiverse yet threatened biomes, the Atlantic Forest. We apply a field-based “taxogenomic” approach, comprising the integration of classical fungal taxonomy and genomic characterization of a sample’s endogenous, associated, and incidental DNA. The data we produced in the field reveal a new species of *Purpureocillium* fungus belonging to the *P. atypicola* group, parasitizing trapdoor spiders, and provide a snapshot of its associated bacterial and fungal microbiota. Molecular, morphological, and ecological data support *P. atypicola* as a complex of cryptic species infecting a variety of ecologically distinct spider species globally. We call for consolidated efforts to accelerate and facilitate the publication of both new species and the characterization of the genomic composition of their associated taxa.

## ﻿Introduction

The scientific documentation of biodiversity is an essential step toward understanding and protecting life on Earth and, as such, is at the core of multiple goals and targets of the Kunming–Montreal Global Biodiversity Framework (Conference of the Parties to the Convention on Biological Diversity [CBD] 2022) and the national policies and actions that arise from it. However, about 86% of terrestrial and 91% of marine species remain scientifically undescribed (Mora et al. 2011). Considering fungi, more than 90% of the 2.5 million species estimated to exist are yet to be described (Niskanen et al. 2023). For arthropod-associated fungal groups, this knowledge shortfall is exceptionally acute.

In naming new species as stable taxonomic entities, their molecular characterization beyond the genetic regions classically used for phylogenetic inference (i.e., a single or a handful of loci obtained through Sanger sequencing) can be critical. Among all species that have been described so far, only a tiny fraction—0.2% of all animals, 0.5% of all flowering plants, and 4.4% of all fungal species—have had their genomes sequenced and publicly released (Hotaling et al. 2021). Unless we increase the taxonomic and genomic characterization of biodiversity—particularly in the tropics—many species may disappear before their biological interactions are even known to science. While species remain unknown, they cannot be considered in conservation policies, and decisions are therefore made with incomplete data, potentially reaching biased conclusions with suboptimal outcomes (Antonelli 2023).

Current barriers to increasing the rate of biodiversity documentation—from genes to taxonomic units—are complex and intertwined (e.g., Meyer et al. 2016). Multiple logistical, ethical, legal, and social challenges need to be overcome (Lewin et al. 2018; Sherkow et al. 2022), with methodological constraints playing a significant part but being perhaps the simplest to address. A key step in the process is the generation of DNA sequence data for any putative new species alongside a reference library for comparisons, a goal envisaged by consortia such as the Earth BioGenome Project (Lewin et al. 2018), the African BioGenome Project (Ebenezer et al. 2022), and the European Reference Genome Atlas (Formenti et al. 2022). While molecular sequence references for taxonomy are most useful if produced from type specimens, to date a mere 21,000 species of fungi have sequence data available from type collections, representing less than 1% of the total estimated fungal diversity (Renner et al. 2023). To address this shortfall, initiatives to sequence both barcoding genes (ITS) and whole fungal genomes are underway. For the barcoding of types, the FunDive project based at the University of Copenhagen stands out as a pan-European initiative uniting collections from 24 countries (https://fun-dive.eu). Efforts in the United Kingdom to sequence genomes of fungal type specimens deposited at the Royal Botanic Gardens, Kew, the Natural History Museum in London, and the Royal Botanic Garden Edinburgh are also currently underway.

In relatively wealthy countries with well-developed research infrastructure, long DNA sequences can be readily produced—as is currently done by the Darwin Tree of Life project in the UK, aiming to sequence whole genomes of all eukaryotic species in the country (The Darwin Tree of Life Project Consortium 2022). When considering the generation of data for identifying and genomically characterizing new species in regions where such infrastructure may not be readily available, real-time DNA sequencing using Oxford Nanopore Technologies (ONT) has been proposed as part of the solution (Parker et al. 2017; Krehenwinkel et al. 2019). Its portability has made it attractive to researchers working in remote locations, with the added advantage of opening doors to more democratized scientific discovery and scientific training with researchers in biodiverse low- and middle-income countries (Pomerantz et al. 2018; Watsa et al. 2020; De Vivo et al. 2022).

Here, we apply in-the-field ONT sequencing to investigate cryptic species that are broadly distributed across arthropod-associated hypocrealean fungi. Based on prior knowledge of existing complexes, we identified and aimed to collect such examples where data are lacking. For example, we searched for *Ophiocordyceps unilateralis* (the zombie-ant fungi), *Gibellula* (infecting and manipulating spiders), *Akanthomyces tuberculatus* (infecting adult moths), and *Purpureocillium atypicola* (infecting spiders worldwide), among others.

Our study was conducted in Brazil’s Atlantic Forest, which is both a global biodiversity hotspot (Morellato and Haddad 2000; Myers et al. 2000) and a darkspot—where many species are yet to be described and mapped (Ondo et al. 2023). To sequence a broad cross-section of the genome, we develop and validate a genome-skimming PCR-free methodology. We use a non-targeted approach to ensure ample data for genomic analyses for this study as well as in the future, e.g., the exploration of gene functions and associated microbiomes. This differentiates our method from other biodiversity assessments involving ONT sequencing in the field, where PCR barcoding of common markers has been applied (16S, CO1; Menegon et al. 2017; CytB, NAHD, ND4; Pomerantz et al. 2018) or longer stretches of ribosomal DNA spanning several genes (Krehenwinkel et al. 2019). This methodology also shortens laboratory time, decreases demand on energy supply, and allows the quantification of endogenous and exogenous sequence data. These aspects of our approach, when applied to other taxa and regions, will widen the possibilities for evolutionary, ecological, functional, and taxonomic investigations across distinct groups of neglected tropical fungi.

## ﻿Materials and methods

### ﻿Field sampling

We carried out a field survey in November 2022 at RPPN Alto da Figueira in the Atlantic Forest of southeast Brazil, a pristine cloud forest reserve that is home to the headquarters of the Atlantic Forest Research and Conservation Alliance (ARAÇÁ)—the flagship project of the Hidden Universe: Biodiversity Foundation (www.hu-b.org). To maximize the chances of finding at least one of the species complexes of interest and to contribute to ongoing research efforts by members of our team, our attention was focused on arthropod-associated fungi, one of the many understudied fungal groups in the region.

We followed the collecting protocol presented by Araújo et al. (2018) and performed a 6-hour survey that consisted of a careful inspection of the soil, leaf litter, shrub leaves, and tree trunks up to ca. 2 m high. The collected specimens and their substrata were carefully cleaned with a soft brush and stored individually in plastic containers, transported to the field base, and examined on the same day. The specimens were photographed individually using a Canon R7 camera equipped with an EF 100 mm macro lens. Voucher numbers were provided and deposited at the Herbarium of the Universidade Federal de Santa Catarina (FLOR) in Brazil.

### ﻿Morphological characterization

To characterize the macromorphological features, we followed the methodology of Araújo et al. (2018), in which specimens are examined using a stereoscopic microscope (Leica EZ4) for macromorphological characterization. The traits investigated consisted of host location (e.g., burrow, leaf, spine, trunk, moss, base of trunk, soil); interaction between fungus and substrate (e.g., presence/absence of attachment structures); ascomatal size, color, position, presence/absence, and characterization of asexual morphs. For micromorphological characterization, free-hand sectioning of the ascoma was performed and mounted on a slide with lactic acid for light microscopy examination (conidiogenous cells, conidia) using an Olympus BX53 equipped with Olympus QColor 3. A minimum of 30 structures were measured per category (e.g., conidiophore and conidia).

### ﻿Molecular methods

We isolated DNA using the MagAttract kit (QIAGEN, Germantown, MD, USA) from the fungal fruiting body and decaying arthropod legs. We performed library preparation and pooling using a Rapid Barcoding Kit with v.9 chemistry (SQK-RBK004; Oxford Nanopore Technologies [ONT], Oxford, UK), and the barcoded pool for each taxon was loaded onto an R9.4.1 SpotOn sequencing flow cell (FLO-MIN106D; ONT, Oxford, UK) using a flow cell priming kit (EXP-FLP002; ONT, Oxford, UK). Sequencing was carried out using a MinION Mk1C.

We mined homologs of known phylogenetically informative loci from our ONT reads and placed them in a phylogenetic framework to support taxonomic identification and description of new species. First, the reads were demultiplexed (Guppy v.6.3.8, ONT), and the adapters were trimmed with Porechop v.0.2.4 (Wick et al. 2017). We used Nanofilt (De Coster et al. 2018) to filter for reads with a minimum Q9 quality score and to crop the leading and trailing 30 bp from each sequence. We evaluated the output with NanoPlot (De Coster and Rademakers 2023). Second, we extracted reads matching phylogenetically informative loci (ITS, LSU, SSU, *TEF*) using Magic-BLAST v.1.5.0 (Boratyn et al. 2019). The selection of these loci was based on Araújo et al. (2022) for the order *Hypocreales*. Due to the considerable per-read error rate attributed to early ONT sequencing versions such as those used here, we further filtered out reads smaller than 200 bp and by an identity percentage >80 to ensure that the reads were correctly assigned to their corresponding loci. Reads for each of the targeted loci were aligned using MAFFT v.7.520 (Katoh and Standley 2013) on a per-sample basis.

We then mined gene sequences for ITS, LSU, SSU, and *TEF* from NCBI for seven specimens of *P. atypicola* from Thailand, the USA, and Japan exhibiting distinct ecological features. Because of the limited availability of representative sequences for ITS, LSU, and SSU for the group and to avoid biases from missing data in maximum likelihood tree inference (Roure et al. 2013), we assembled a densely sampled dataset using *TEF* sequences. To test for copy variation in the *TEF* gene in the sequenced specimen, we enabled the placement of multiple reads matching the *TEF* gene (Suppl. material 1: table S3).

We inferred a maximum likelihood tree using RAxML v.8.2.4 (Stamatakis 2014). We also produced consensus tree networks from the bootstrap tree replicates derived from RAxML to better depict relationships among higher taxonomic ranges, using the software SplitsTree5 (Huson and Bryant 2005). We employed the GTRGAMMA substitution model and carried out 500 bootstrap replicates. Visualization and graphic adjustments were performed in Dendroscope (Huson and Scornavacca 2012) and further edited in Adobe Illustrator.

To assess the proportion of endogenous DNA retrieved from our specimen, we first filtered ONT reads with a quality inferior to Q10 and a length of 300 bp. The retained reads were mapped against the reference genome of *Purpureocillium takamizusanense* (GCA_022605165.1—a close relative to our specimen) and its corresponding coding DNA sequence using BLAST+ v.2.10 (Camacho et al. 2009) and the following parameters: evalue 0.001, -max_target_seqs 10, and -max_hsps 10. To further remove potentially spurious alignments, the resulting hits were filtered by retaining matches with a minimum alignment length of 300 and a percentage identity of 80. Chromosome- and gene-level coverage were calculated from unique hits.

Due to the inherently higher error rate of single-molecule sequencing when compared to simultaneous sequencing of local clusters of the same molecule (Dohm et al. 2020), we sought to benchmark the use of our non-amplified ONT-generated gene sequences used for the phylogenies. We did this by performing Sanger sequencing for two of the genes, LSU and SSU, using these as assumed correct representations of the true DNA sequence. ITS and *TEF* were not used, as these were not successfully amplified via PCR. Nonetheless, we assume that the genes used for comparison are an adequate general proxy for quality assurance in ONT reads. The two loci were sequenced following the protocols presented by Araújo et al. (2018).

We assumed that read accuracy for the ONT v.9 chemistry used here (available to us at the time of fieldwork) should be around 90% (Wick et al. 2019). To evaluate which of our genes showed sufficient coverage with a tolerable error rate, we simulated batches of DNA reads with different final coverages with respect to the target sequence (Suppl. material 1). We employed NanoSim (Yang et al. 2017), which models the base-calling errors of ONT reads to inform the simulation of sequences with similar error characteristics. For the characterization step, in which the model requires a training set, we used as input our trimmed, minimum Q9 reads that had been mapped to the respective genes in the Magic-BLAST step. We set the Sanger-sequenced gene as the reference. To attain comparable coverage at the ends of our simulated reads (i.e., to generate artificial ONT long reads that extend beyond our reference gene sequence), we required flanking sequences. Appropriate high-quality reads of sufficient similarity were determined by running BLAST searches of a few ONT reads that extended beyond the gene region, identifying *P. takamizusanense* strain PT3 chromosome 12 (GenBank CP086365.1) as the closest match. We used this chromosome to add 100 bp of artificial high-similarity flanking region to either end of the reference read. Importantly, this was only used to foster generation of long reads and hence coverage, and not to score bases past the limits of the target gene. We then ran NanoSim’s characterization step in “genome” mode with default settings.

For the simulation step, we used the error profile output from the characterization step in conjunction with the flanking region-enhanced reference genome, setting guppy-flipflop base-calling and minimum read length to the 95^th^ quantile of the fragments’ respective read length distributions obtained in the experimental data. We reproduced this for different scenarios of sequence depth (2, 5, 10, 25, 50, and 75), replicating the experiment in triplicate. We examined the output as consensus sequences in Geneious v.2023.2.1 and summarized the difference in terms of polymorphisms observed between the Sanger reference and the consensus of the simulated reads relative to the Sanger reference gene. To compare the outcome with real measurements of depth in our reads mapped to the LSU and SSU, we mapped these to the respective Sanger sequences using minimap2 (Li 2018).

### ﻿Conservation status assessment

Conservation status was determined following the International Union for Conservation of Nature criteria (IUCN Standards and Petitions Committee 2022), using all available data to assess the species. When relevant, specific adaptations suggested for fungal species by Dahlberg and Mueller (Dahlberg and Mueller 2011) were followed.

## ﻿Results

Our rapid biodiversity survey in Brazil’s Atlantic Forest (one day of sampling by one mycologist), followed by DNA extraction, sequencing, and analyses alongside a morphological evaluation, resulted in the recovery of five specimens of arthropod-pathogenic fungi, including one previously undescribed species from the genus *Purpureocillium* (*Hypocreales*: *Ophiocordycipitaceae*). The specimen was infecting a trapdoor spider buried in the ground and resembled a synnematous (producing a stalk) *P. atypicola*, a relatively common spider parasite in the USA, Japan, and Thailand (Yasuda 1915; Kobayasi and Shimizu 1977; Hywel-Jones and Sivichai 1995). Only the apical part of the spore-producing structure (synnema/fruiting body) was visible emerging from a hole in the ground. Upon closer examination, we found that the hole was a trapdoor spider burrow containing a spider at the bottom, mummified by white fungal mycelia and a cylindrical purple synnema erupting from its cephalothorax. The spider host possibly belongs to the *Ctenizidae*, a family of trapdoor spiders that distinctively construct burrows in the ground covered by a mechanism that is quickly opened when the spider grabs prey passing by. These features observed while still in the field strongly suggested that the specimen was a member of the *P. atypicola* group, which fell within one of our focal cryptic groups of interest.

We here propose the new species primarily on the grounds of its distinctive morphology, life history, ecology, and distribution, with molecular phylogeny providing additional independent support for this taxonomic conclusion.

### ﻿Taxonomy

#### ﻿ Purpureocillium atlanticum

Taxon classificationAnimaliaHypocrealesOphiocordycipitaceae

Araújo, Martins-Cunha, Antonelli & Drechsler-Santos sp. nov.

C16638E6-5A83-5817-A4BF-6A61493C926D

853794

Fig. 1

##### Diagnosis.

Macro- and micromorphological structures divergent from the type collected by A. Yasuda and characterized by Petch (1939): smaller clava (2 cm long versus 6 cm), larger phialides (4–8 × 2.4–4.3 µm versus 4–5 × 2 µm), and larger and cylindrical conidia (3.5–6.6 × 1.6–2.7 µm versus 4–6 × 1.5–2 µm).

##### Description.

**Figure 1.**
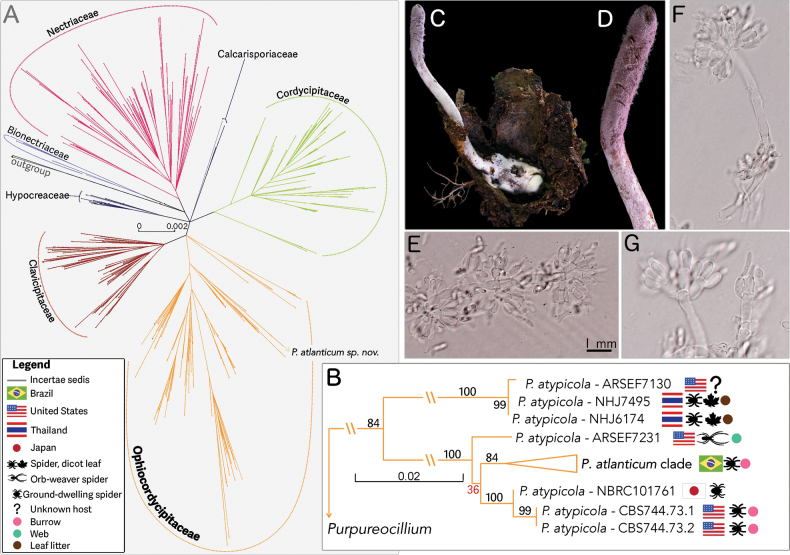
Phylogeny of hypocrealean fungi (A), highlighting the *Purpureocillium atypicola* complex (B) and morphology of the new species, *P. atlanticum* (C–G). A Maximum likelihood (ML) tree of the order *Hypocreales*, with *P. atlanticum* highlighted, obtained from a dataset of the *TEF* gene. B *Purpureocillium atlanticum* sp. nov. is indicated by bold font. Details about the country, death location, and host ecology are indicated in the legend at the bottom-left corner. C Trapdoor spider inside its (dissected) burrow covered by white fungal mycelium and the cylindrical purple synnema arising from the cephalothorax. D Close-up of the apical spore-producing region of the fruiting body. F, G Conidiophores forming dense clusters bearing phialides (spore-producing cells) and conidia at the tips.

The fungus infects trapdoor spiders buried in the forest floor inside their burrows, covering the spider almost completely with soft, cotton-white mycelium. ***Stroma*** single, cylindrical to clavate, slightly sinuous, arising from the host and growing towards the burrow opening, white at the base turning purple and pruinose in the terminal fertile part upon maturity and conidia production; up to 2 cm long × 2–3.5 mm diameter when fresh (1.5 cm long × 1–2.5 mm diameter when dried). ***Conidiophores*** abundant, densely grouped, hyaline, smooth-walled, with intercalary and/or terminal fertile portion (ca. up to 6 clusters per conidiophore; Fig. 1F, G), 140 µm long × 2.6 µm wide, typically enlarging up to 4.5 µm where metulae emerge; metulae (typically up to 5 around a septum, or terminal) and phialides (no more than 10 per metulae) in rosetoid arrangement attached to the septum or terminally on the conidiophore. ***Metulae*** typically formed around a septum or terminally (Fig. 1F, G), clavate to obovoid, hyaline, (4.5–) 4.7–7.5 (–8.7) × (2.44–) 2.7–4 (–4.53) µm, borne directly on main hyphae along the conidiophore, with 4–10 phialides. ***Phialides*** ellipsoid to obovoid, hyaline, (4.25–) 5–7.5 (–8.11) × (2.38–)2.67–3.89(–4.28) µm, neck absent. ***Conidia*** abundant, cylindrical, smooth, and thin walled, hyaline, with characteristic 1–2 droplets inside, (3.49–)4.3–6.37(–6.6) × (1.6–)1.68–2.44(–2.7) µm. ***Sexual morph*** not observed.

##### Etymology.

The specific epithet refers to the Atlantic Forest of eastern South America, one of the world’s most biodiverse and threatened biomes.

##### Holotype.

Rio de Janeiro: Nova Friburgo: RPPN Alto da Figueira (22°22'31.0"S, 42°49'44.0"W; c. 1,400 m a.s.l.), 22 Nov 2022, *J.P.M. Araújo., A. Perrigo & A. Antonelli* (FLOR73173), on trapdoor spider. FLOR = Herbarium of the Universidade Federal de Santa Catarina, Brazil.

GenBank accession numbers (holotype FLOR 73173): SSU - PX571242; LSU - PX571243; *TEF*- PX548866.

##### Notes.

This species is related to *P. atypicola*, which was described from specimens found on a spider in Japan. The morphological, ecological, geographical, and phylogenetic diversity shown within the *P. atypicola* clade suggests it is a species complex. The materials examined from the group exhibit a wide range of distinct morphological and ecological features and include specimens from different parts of the world (*Nomuraea atypicola* (Samson 1974; Hywel-Jones and Sivichai 1995); *Spicaria atypicola* (Petch 1939)).

##### Conservation status and distribution.

Data Deficient (DD). *Purpureocillium atlanticum* sp. nov. is only known from one site in Brazil’s Atlantic Forest. It is likely that the species also occurs in other similar old-growth and well-preserved sites. Our team has collected sexual stages of “*P. atypicola*” in the Amazon before, but since the clade is now treated as a complex of species, more detailed studies will be conducted on these previously collected *P. atypicola*-like samples. The cloud forests where it was found are restricted to areas above an elevation of 1,000 m above sea level, and since these areas are highly reduced, fragmented, and dependent on specific microclimatic conditions (Bruijnzeel et al. 2011; Oliveira et al. 2014), the species might be susceptible to threats caused by climate change and further habitat reduction and fragmentation (Salazar et al. 2007; Williams et al. 2007; Goldsmith et al. 2013; Gotsch et al. 2014; Pompeu et al. 2014; Helmer et al. 2019).

### ﻿Molecular results

After filtering out low-quality reads, ~208 million bases were retained (Suppl. material 1: table S1, fig. S2, Extended methods and results). Read mapping against the reference genome of *P. takamizusanense* yielded 81 million base pairs, covering 2,115 genes (out of 10,921 annotated genes) with a sequencing depth ranging from 1–219× (Suppl. material 1: table S2). Read mapping of unfiltered sequence data against three widely used genes for phylogenetic tree inference in fungi (LSU, SSU, *TEF*) recovered sequencing depths of ~150× to ~400× for the lowest quality reads (Q7) and 1× to ~20× for the highest quality reads (Q13) (Suppl. material 1: fig. S3). Additionally, simulations of long reads with varying base-calling error rates and coverages for LSU and SSU indicated that error rates were exceedingly high at very low coverages (1–5×) but decreased sharply, becoming negligible (<1%) at sequencing depths of 10× or higher (Suppl. material 1: fig. S1). This supports the reliability of our preliminary phylogenetic results drawn from *TEF*, SSU, and LSU gene fragments sequenced using v.9 ONT chemistry in the field (respective depths: 160, 234, 394).

We were able to confirm, based on molecular data—still in the field, four days after collection—that one of the fungi we found belongs to the genus *Purpureocillium*, with the closest BLAST results matching *P. atypicola* (ITS = > 97% and *TEF* = > 96%). Despite its broad distribution and ecological breadth, the genus includes only eleven species described so far, i.e., *P. aranea*, *P. atypicola*, *P. hyophilae*, *P. lavendulum*, *P. lilacinum*, *P. roseum*, *P. sinense*, *P. sodanum*, *P. takamizusanense*, *P. tiankengense*, and *P. zongqii* (www.mycobank.org; www.speciesfungorum.org). These fungi exhibit a variety of ecologies and include species associated with soil, decaying vegetation, insects, spiders, nematodes, and even air contaminants that can cause infection in immunocompromised humans and other vertebrates (Luangsa-Ard et al. 2011; Calvillo-Medina et al. 2021; Chen et al. 2025). Read mapping against the reference genome of *P. takamizusanense* recovered 10.1 Mb of endogenous DNA (genome size of *P. takamizusanense* = 35.5 Mbases) and 8.1 Mb from 2,115 genes (out of 10,859 annotated genes in *P. takamizusanense*) (Suppl. material 1: table S2).

### ﻿Associated and incidental microbial species

Our PCR-free method allowed a qualitative assessment of the exogenous (incidental and potentially associated) microbial diversity of the holotype samples, which we conducted on the two DNA samples (fungus and host) that yielded the new species, through exhaustive BLAST searches against the entire nuclear database at NCBI.

**Figure 2.**
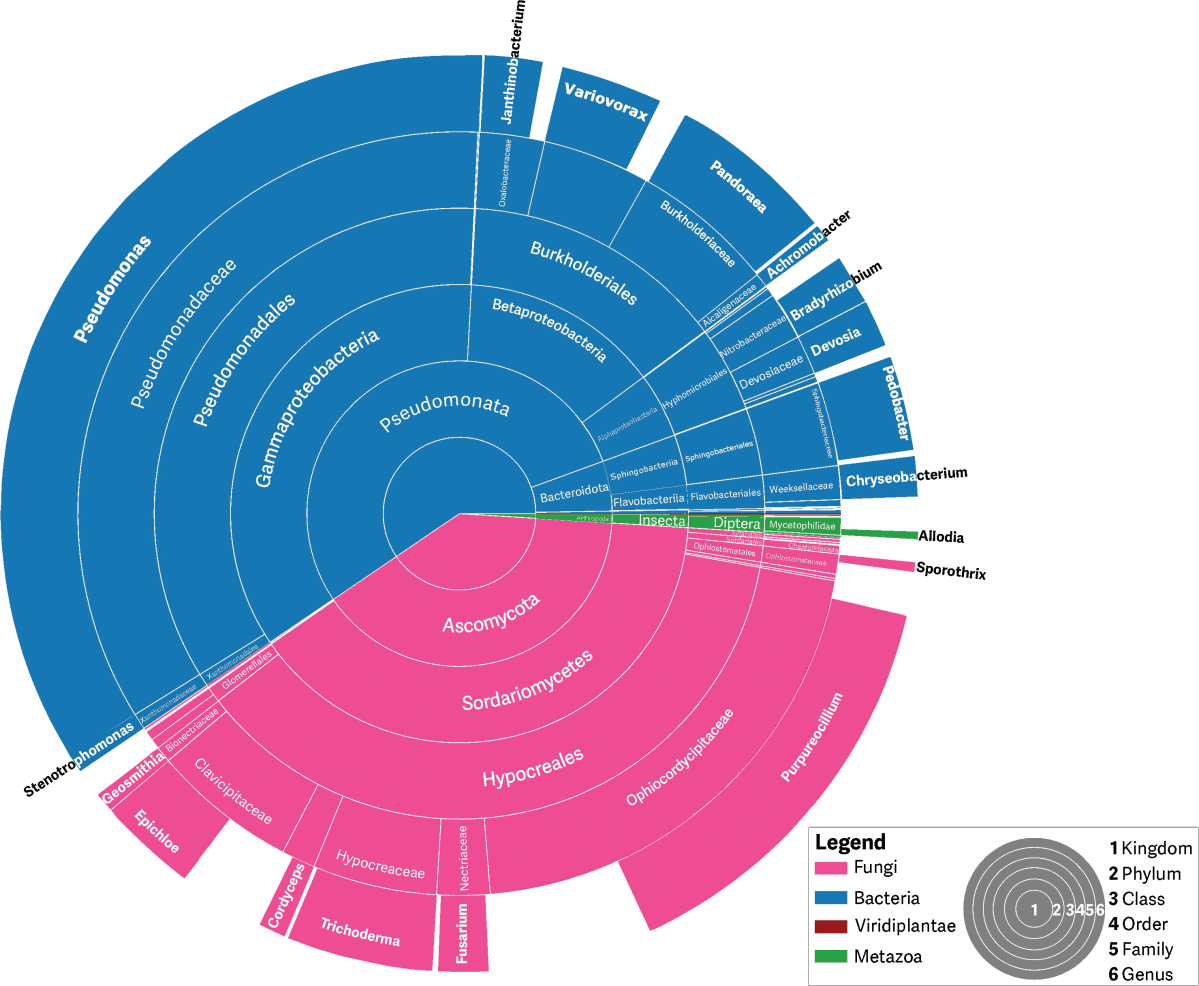
Representation of BLAST hits showing sequences on the NCBI database of highest similarity to Oxford Nanopore Technology read data and capturing a proportion of the exogenous (associated and incidental) diversity of *P. atlanticum*. The NCBI nr database was used for this search, and each hit was classified into the closest identifiable taxon.

We found a predominance of bacteria belonging to the gram-negative phylum Pseudomonadota, which comprises pathogenic and free-living aerobic taxa with diverse ecological roles, including *Pseudomonas*, *Variovorax*, and *Pandoraea*, as well as DNA from other fungal genera such as *Trichoderma* and *Epichloë* that have been reported in South America but are understudied. Additionally, a small proportion of our ONT sequences matched genomic regions of the fly *Allodia* (*Mycetophilidae*; Fig. 2), suggesting the presence of arthropod DNA. Whether this DNA belongs to the spider trapdoor or the host of *P. atlanticum* remains to be assessed, since there are very few reference sequences of those species in NCBI.

## ﻿Discussion

### ﻿Disentangling fungal biodiversity: *P. atypicola* as a species complex

**Figure 3.**
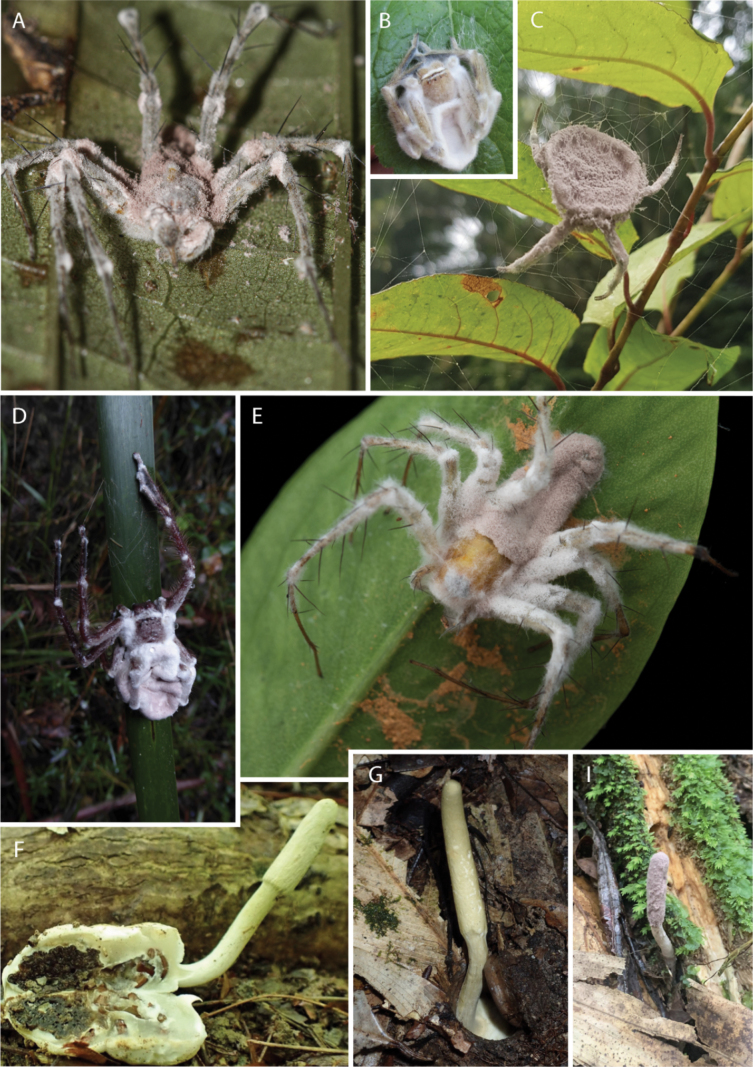
The cosmopolitan *Purpureocillium atypicola* complex exhibits a variety of morphologies (e.g., sessile anamorph (A–E), stalked sexual morph (F–G), and asexual morph (I, *P. atlanticum*)), spider hosts, ecologies, and death locations, indicating cryptic species diversity.

Our study, the most comprehensive phylogenetic analysis of the *P. atypicola* clade to date (Fig. 1B), representing its broad diversity of morphological and ecological features (Fig. 3), builds on previous studies on the family *Ophiocordycipitaceae* and the genus *Purpureocillium* based on traditional PCR-based methods such as Sanger sequencing (Luangsa-Ard et al. 2011; Quandt et al. 2014; Araújo et al. 2022). We propose that *P. atypicola* is not a single species—as it has been considered for more than a century (Yasuda 1915)—but rather a diverse, widespread, and neglected species complex comprising several undescribed fungal species that are morphologically, ecologically, and genetically distinct. These groups play different roles in the habitats they occupy, infect distinct hosts, exhibit distinct morphological features, and likely contain a wealth of potentially useful secondary metabolites (Chen and Hu 2022). This makes their genomic characterization of particular interest for bioprospecting purposes.

### ﻿Historical background

Fungal lineages that evolved the ability to infect, establish within, and sporulate from spiders can be found scattered among the families *Cordycipitaceae* and *Ophiocordycipitaceae* within the order *Hypocreales*. The cordycipitaceous genus *Gibellula* is notably the most commonly found and diverse fungal genus among spider-associated fungi (Shrestha et al. 2019; Mendes-Pereira et al. 2022, 2023; Evans et al. 2025). Furthermore, other genera are also known as spider parasites, such as *Arachnidicola*, *Cordyceps* (*C. caloceroides*), *Hevansia*, *Parahevansia*, *Jenniferia*, and *Polystromomyces* (Mongkolsamrit et al. 2022). In *Ophiocordycipitaceae*, spider parasites are rare, represented by a small number of species in *Ophiocordyceps* (Samson and Evans 1975; Nyffeler and Hywel-Jones 2024; Yang et al. 2025), and by *Purpureocillium* (*P. atypicola* complex).

*Purpureocillium atypicola* was originally recorded in Japan by Yasuda (1894). He misapplied the name *Isaria arachnophile* to this fungus and provided a short description in Japanese. Later, he noticed his misapplication and re-described it as a new species, *Isaria atypicola*, but again solely in Japanese (Yasuda 1915). In 1917, he presented the characteristics of this fungus in a European language (German) for the first time. No vouchered specimen was cited in either of his papers, but his description indicated that the specimen was collected at the Koishikawa Botanical Garden in Tokyo (“botanischen Garten der Tokyo Kaiserlichen Universiaat, Juli 1910,” Yasuda 1917). According to the current ICN (Shenzhen Code), if only one specimen was specified in a pre-1935 publication, it is automatically regarded as the type. Therefore, the referred specimen collected in “Tokyo” (TNS-F-203218 collected in 1915) could be regarded as the holotype, although it was never designated as such. Because the holotype specimen (TNS-F-203218) is too old and degraded for DNA extraction and morphological assessment, it would be possible to designate another newly collected specimen as an epitype. Because it is highly possible that Yasuda’s specimen was collected in Koishikawa Botanical Garden in Tokyo, future work is planned to address the collection of specimens and designation of the epitype for *P. atypicola*.

### ﻿Host association, ecology, and morphological features

The original host of *P. atypicola* was described as *Atypus karschi* (hence the original epithet “atypicolum”; Yasuda 1915) and later corrected as Latouchia (Kishinoyeus) typica (Petch 1939), which only occurs in Asia (World Spider Catalog 2023). Therefore, our newly described Brazilian *P. atlanticum* infects a species of trapdoor spider belonging to a distinct family from that of *P. atypicola* sensu stricto, likely *Ctenizidae*. So far, four host ecologies are recorded for spiders infected by *P. atypicola* sensu lato (s.l.): web (orb-weaver spiders), burrow (trapdoor spiders), leaf litter/boulders (ground-dwelling), and underside of leaves (foliicolous) (Fig. 3). This means that lineages within the *P. atypicola* clade occupy a variety of niches in the forests they inhabit. Interestingly, distinct lineages within the *P. atypicola* complex infecting ecologically distinct spiders exhibit morphological adaptations that seem to relate to host ecology. For example, *P. atypicola* s.l. infecting orb-weaving spiders (e.g., *Nephila* and *Argiope*) invariably exhibit mononematous conidiophores (sporulating directly from the host body; Fig. 3A–E), while those infecting ground-dwelling spiders—notably trapdoor spiders—are consistently synnematous (produce a stalk; Fig. 3F–H) because the fungus needs to project the fruiting body outside the burrow to disperse spores, either at its sexual stage (*Cordyceps cylindrica*-like; Fig. 3F, G) or asexual stage (e.g., *P. atlanticum*; Fig. 3H). The connection between the sexual state (formerly *C. cylindrica*) and the *P. atypicola* asexual state was suggested by Petch (1939) but only demonstrated experimentally decades later by Evans (1982), when ascospore isolations (sexual spores from *C. cylindrica*) from Amazonian specimens produced the purple *P. atypicola* asexual morph in culture.

**Figure 4.**
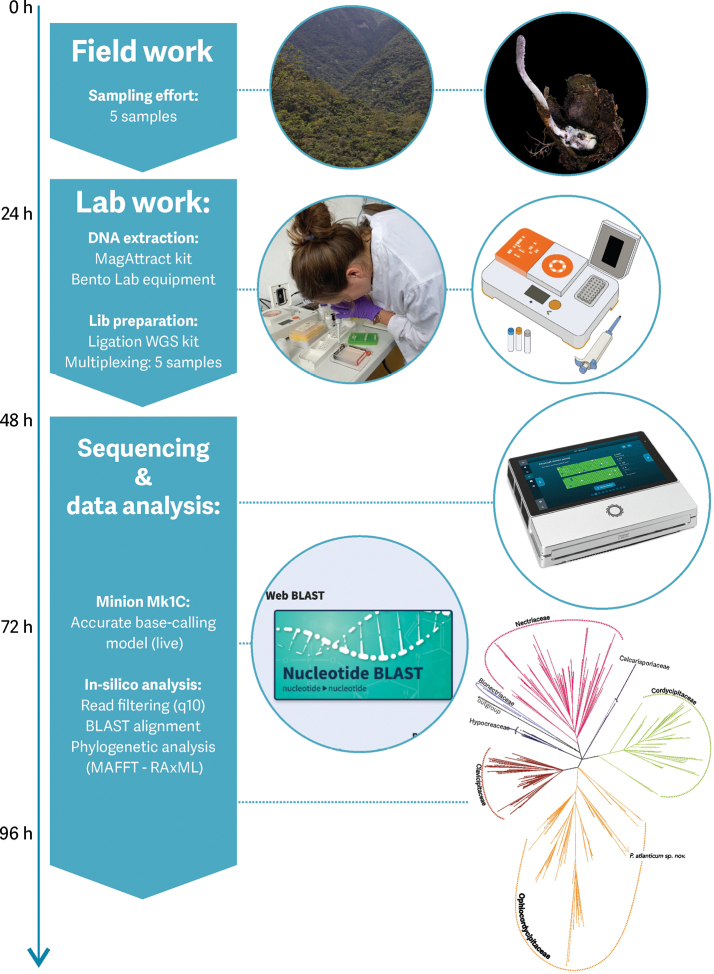
Workflow summarizing the various steps from fieldwork to the production and analysis of genomic data (see text for details). For this study, five samples were collected in Brazil’s Atlantic Forest, including one specimen of “*Purpureocillium atypicola*” (*P. atlanticum* sp. nov., FLOR73173). The laboratory work involved field-based DNA extraction and library preparation. The sequencing and data analysis were carried out using a MinION Mk1C. The flowchart depicts an approximate timeline (in hours). Both the software and the DNA reference sequences for the taxonomic groups to be surveyed can be downloaded beforehand to enable completion of the full pipeline without the need for internet connection.

*Purpureocillium atypicola* s.l. has a global distribution and has been recorded in the Neotropics (Petch 1939; Mains 1954; Evans 1982), Africa (Samson 1974; Samson and Evans 1977; Rong and Grobbellar 1998), and Asia (Yasuda 1894; Kobayasi 1941; Kobayasi and Shimizu 1983; Hywel-Jones and Sivichai 1996), exhibiting striking morphological, ecological, and molecular diversity as shown in Figs 1, 4. Therefore, our results provide clear morphological, ecological, and molecular data to propose *P. atypicola* as a complex of species, confirming the suspicions raised by Evans (2013). Further studies on the phylogeny, morphology, and ecology of the *P. atypicola* complex are needed to understand its true taxonomic and genomic diversity, evolution, and functional morphology, and its potential to harbor medicinally important metabolites and other useful genes (Chen and Hu 2022).

### ﻿Methodological considerations

The taxonomic description of new species using ONT methods has been performed in the past, but it has often been limited by the reduced data output of flow cells, high error rates, and the complexity of eukaryotic genomes, leading researchers to opt for single- or multiple-gene PCR strategies targeting barcoding genes (Araújo et al. 2018; Pomerantz et al. 2018; Krehenwinkel et al. 2019; Chang et al. 2020; Pomerantz et al. 2022). Considering improvements in the accuracy of the newest versions of ONT chemistry, there is an exciting and largely unexplored niche for biodiversity research.

Our experimental genome-skimming approach, combined with detailed taxonomic examinations, was successful in supporting species discovery and partial genomic characterization within just a few days. While bypassing the PCR step permitted access to many more genomic regions, one associated limitation was the broad fragment size distribution of the eukaryotic gDNA input libraries. The characteristic minor proportion of longer reads in each sample increased the risk of dominance of certain barcodes—and hence certain samples—during the initiation of sequencing on the flow cell (Suppl. material 1). DNA yield was also a limiting factor in this study, and this aspect could be improved by the development of better protocols for DNA extraction in the field for various taxonomic groups.

Another potential limitation is that while our approach can characterize genomic regions beyond traditional barcodes, the throughput of read data needed to accurately correct base-calling errors inherent in ONT sequencing can be insufficient (Koren et al. 2017), limiting the completeness of representations of eukaryotic genomes with sufficient accuracy for certain purposes (such as *de novo* genome assembly). Although we did not have access to the latest ONT chemistry for this study, our simulations on the raw ONT data showed that upon reaching a sequencing depth of 10, the error rate dropped to negligible levels (Suppl. material 1), thus reducing potential biases in phylogenetic inferences—in line with studies assessing fungal identification in a clinical context that compared Sanger and ONT sequencing (Morrison et al. 2020).

Despite these limitations, we obtained sufficiently robust data to allow accurate taxonomic placement of our samples into the right species cluster, taking a leap forward from barcoding-based approaches (Srivathsan et al. 2018) to simultaneously characterize the partial genome of the new fungal species and unveil key features of its associated microbiota. These taxogenomic results advance our knowledge of the natural history of a highly diverse and understudied group.

### ﻿Advancing taxogenomics

We have shown how a relatively simple analytical workflow, using equipment that is becoming affordable to many research organizations and scientific projects, can support the joint characterization of previously unknown taxonomic and genomic biodiversity—even for a poorly studied group of organisms. This approach is most useful in biodiverse regions lacking the necessary infrastructure for other forms of DNA sequencing. While the sequences obtained from ONT may contain more errors than classical Sanger technology, which requires outsourcing of the sequencing, we show that they are sufficient for a reliable phylogenetic placement to support the description of new taxa to science already in the field, along with their partial (and in the future potentially full) genomes, and to also characterize associated and incidental microbial DNA. The use of ONT, or any other molecular method for taxonomic purposes, is optimal when integrated with other sources of data (e.g., morphological, ecological, ethological), although for many species-rich and understudied groups, such associated data may take many years to be documented and collated.

Additional benefits of a field-based approach (“taking the lab to the field”) include opportunities for capacity strengthening of local students and researchers through collaboration and associated bi-directional knowledge transfer; a simplified legal framework, since no biological samples need to be exported; and the deposition of samples at local natural history collections, facilitating their access in the country of origin for further research and conservation.

## ﻿Conclusion

Describing and mapping the world’s biodiversity is a major scientific and societal goal. At the current pace, it may take between 750 and 1,000 years for a comprehensive inventory of all fungal species to be complete (Niskanen et al. 2023). To accelerate and improve this process, we need to explore what is hindering our community and how to improve and accelerate taxonomic discovery. Now that technology can no longer be considered a major bottleneck, and laboratory costs are increasingly affordable for research projects in the Global South, we highlight three areas that need attention: i) greater training and support is needed for students and other researchers to generate and process taxogenomic data, particularly in biodiverse low- and middle-income countries; ii) directed surveys focusing on dark taxa, i.e., those bearing immense potential for taxonomic and biotechnological discoveries (for example, arthropod-pathogenic fungi), should be intensified; and iii) there needs to be a concerted effort to generate genomic references and morphological trait information from authoritatively identified material.

Addressing these three challenges and supporting regions and initiatives that need the most attention, including global biodiversity darkspots (Ondo et al. 2023), will be key to implementation of the Kunming–Montreal Global Biodiversity Framework and will unlock a wealth of information on the potential uses of biodiversity for humankind and nature-based solutions to the biodiversity and climate crises.

We hope that our study will not only serve as a basis for other similar taxogenomic assessments in megadiverse regions but also inspire students and professionals to explore innovative ways to accelerate the documentation of our planet’s biodiversity, thereby contributing to its conservation and sustainable use.

## Supplementary Material

XML Treatment for Purpureocillium atlanticum

## ﻿Additional information

### Use of AI

No use of AI was reported.

### Adherence to national and international regulations

All the fungal strains used in this study have been legally obtained, respecting the Convention on Biological Diversity (Rio Convention).

### Author ORCIDs

João P. M. Araújo https://orcid.org/0000-0003-2529-7691

Natalia A. S. Przelomska https://orcid.org/0000-0001-9207-4565

Rhian J. Smith https://orcid.org/0000-0003-2836-0246

Elisandro R. Drechsler-Santos https://orcid.org/0000-0002-3702-8715

Genivaldo Alves-Silva https://orcid.org/0000-0002-8142-6665

Kelmer Martins-Cunha https://orcid.org/0000-0002-0545-5966

Tsuyoshi Hosoya https://orcid.org/0000-0001-5360-5677

Janet J. Luangsa-ard https://orcid.org/0000-0001-6801-2145

Allison Perrigo https://orcid.org/0000-0002-6565-6305

Mar Repullés https://orcid.org/0009-0006-9195-0379

Pável Matos-Maraví https://orcid.org/0000-0002-2885-4919

Oscar A. Pérez-Escobar https://orcid.org/0000-0001-9166-2410

Alexandre Antonelli https://orcid.org/0000-0003-1842-9297

### Data availability

The ONT fastq sequences of *Purpureocillium atlanticum*, the bootstrap replicates of the Hypocreales phylogeny and the supplemental table S3 are available at https://figshare.com/s/17e1381832ba618b9051, GenBank accession numbers at https://doi.org/10.6084/m9.figshare.30675077.v1.

## Supplementary materials

Supplementary material 1 Additional information Data type docx https://binary.pensoft.net/file/1490607This dataset is made available under the Open Database License (http://opendatacommons.org/licenses/odbl/1.0/). The Open Database License (ODbL) is a license agreement intended to allow users to freely share, modify, and use this Dataset while maintaining this same freedom for others, provided that the original source and author(s) are credited.João P. M. Araújo, Natalia A. S. Przelomska, Rhian J. Smith, Elisandro R. Drechsler-Santos, Genivaldo Alves-Silva, Kelmer Martins-Cunha, Tsuyoshi Hosoya, Janet J. Luangsa-ard, Allison Perrigo, Mar Repullés, Pável Matos-Maraví, Roseina Woods, Oscar A. Pérez-Escobar, Alexandre Antonelli
