# Supplementary material for: ﻿A new species of Purpureocillium (Ophiocordycipitaceae) fungus parasitizing trapdoor spiders in Brazil’s Atlantic Forest and its associated microbiome revealed through in situ “taxogenomics”
Source: IMA Fungus. 2025 Dec 12;16:e168534. doi: 10.3897/imafungus.16.168534 (PMC12717516; doi:10.3897/imafungus.16.168534)
Supplement: Supplementary material 1 — Additional information [file imafungus-16-e168534-s001.docx]

**Supplementary Materials for: A new species of *Purpureocillium* fungus parasitizing trapdoor spiders in Brazil’s Atlantic Forest and its associated microbiome revealed through in situ “taxogenomics”**

João P. M. Araújo^1,2^, Natalia A. S. Przelomska^2,3^, Rhian J. Smith^2^, Elisandro R. Drechsler-Santos^4^, Genivaldo Alves-Silva^4^, Kelmer Martins-Cunha^4^, Tsuyoshi Hosoya^5^, Janet J. Luangsa-ard^6^, Allison Perrigo^7,8^, Mar Repullés^9,10^, Pável Matos-Maraví^9^, Roseina Woods^2^, Oscar A. Pérez-Escobar^2^*, Alexandre Antonelli^2,8,11,12^*

1 Natural History Museum of Denmark, University of Copenhagen, Copenhagen, Denmark

2 Royal Botanic Gardens, Kew, Richmond, Surrey, UK

3 School of the Environment and Life Sciences, University of Portsmouth, Portsmouth, UK

4 MIND.Funga/MICOLAB, Botany Department, Santa Catarina Federal University, Florianopolis, Brazil

5 Department of Botany, Division of Fungi and Algae, National Museum of Nature and Science, Tokyo, Japan

6 National Center for Genetic Engineering and Biotechnology (BIOTEC), Thailand Science Park, Pathum Thani, Thailand

7 Lund University Botanical Garden, Lund University, Lund, Sweden

8 Gothenburg Global Biodiversity Centre, Department of Biological and Environmental Sciences, University of Gothenburg, Gothenburg, Sweden

9 Biology Centre of the Czech Academy of Sciences, Institute of Entomology, České Budějovice, Czech Republic

10 Faculty of Science, University of South Bohemia, České Budějovice, Czech Republic

11 Department of Biology, University of Oxford, Oxford, UK

12 Antonelli Foundation for Biodiversity Research and Conservation, Nova Friburgo, Brazil

***Corresponding authors:** [jaraujo@snm.ku.dk](mailto:jaraujo@snm.ku.dk); [a.antonelli@kew.org](mailto:a.antonelli@kew.org); [o.perezescobar@kew.org](mailto:o.perezescobar@kew.org)

**^†^ Joint senior authors.**

Extended molecular methods and results

We applied a sterile scalpel to chop fungal tissue into small pieces, using approximately 0.5 cm^3^ of the fruiting body, and an equivalent amount of tissue from the infested insect legs. To obtain DNA from each sample, we used a MagAttract Kit for extraction of high molecular weight DNA (QIAGEN, Germantown, MD, USA), following the manufacturer’s protocol for fresh tissue. We quantified the extraction and used 400 ng of total DNA for the following steps.

Genomic library preparations were performed using a Rapid Barcoding Kit with v.9 chemistry (SQK-RBK004; Oxford Nanopore Technologies [ONT], Oxford, UK), adding fragmentation mix with a unique barcode to each individual sample. The libraries were then pooled by taxonomic group and prepared for loading onto two R9.4.1 SpotOn sequencing flow cells (FLO-MIN106D; ONT, Oxford, UK) using a flow cell priming kit (EXP-FLP002; ONT, Oxford, UK). Sequencing was carried out using a MinION Mk1C, where FASTQ files were generated using the live “super accurate base calling model” implemented in the ONT software MinKNOW. The de-multiplexed reads were then trimmed for adapters and any contaminating barcode sequences using Porechop v.0.2.4 (Wick et al. 2017). Sanger sequencing of the LSU and nrSSU loci was conducted following the PCR protocol of Araújo et al. (2018).

We examined the quality of the resulting adapter-trimmed reads using FastQC (Andrews 2010) and on this basis trimmed 30bp from the leading end of each sequence and filtered for a minimum read quality score of 9 using Nanofilt (De Coster et al. 2018). Around 208 million bases were produced for the fungal libraries (Table S1, Fig. S2).

We characterised the exogenous DNA content of the ONT read data produced for *Purpureocillium atlanticum* by conducting exhaustive BLAST searches against the standard non-redundant “nr” database of NCBI using BLAST+ v.2.10 (Camacho et al. 2009) and the following parameters: *-evalue* 0.01, -*max_target_seqs* 100, and -*max_hsps* 10. To increase the accuracy of our search, we excluded any results with a bitScore value smaller than 1,000 and queried sequences aligned by less than 100 bases against the subject. The taxonomic backbone of the remaining BLAST hits was retrieved from NCBI using the program efetch of Entrez v.14.4 (Kans 2024). We then estimated the taxonomic diversity in endogenous and exogenous DNA reads by counting the number of sequences matching a particular genus and their corresponding taxonomic hierarchy, up to kingdom level. Sunburst plots were prepared using the *count_to_sunburst* function of the plotme library in R.

To mine standard genes that are used in DNA barcoding and phylogenetics from the ONT data, we used an extensive list of sequences from many species in the Hypocreales (Araújo et al. 2022) for the following housekeeping genes: ITS (internal transcribed spacer), SSU (ribosomal small subunit), LSU (ribosomal large subunit), *TEF* (translation elongation factor)(Table S3). We downloaded the sequences from the NCBI repository and created a BLAST database. We then used magicBLAST v.1.5.0 (Boratyn et al. 2019) with default settings to extract sequences from our raw data which exhibit high similarity to these genes (Fig. S3). We used samtools v.1.10.2 (Li et al. 2009) to convert our quality filtered Nanopore FASTQ reads to FASTA sequences. Each of these loci was aligned individually using Mafft and the alignment strategy (v7.520; Katoh and Standley 2013). To ensure that the reads were correctly assigned to their corresponding barcodes and loci, we performed extra steps of filtering the reads by size and by genetic distances.

For the phylogenetic analyses, we combined two copies of the *TEF* gene for the sequenced specimen into a dataset composed of 929 bp and 326 samples of fungi samples from Hypocreales (Table S3).

We conducted a maximum likelihood phylogenetic analysis from the fungal dataset using RAxML v.8.2.4 (Stamatakis 2014), the GTRGAMMA model of nucleotide substitution, and 500 rapid bootstraps replicates. To better depict poorly supported relationship at the order level, we also computed a consensus network using the 500 bootstrap replicates and SplitsTree v.4.0, with a threshold value of 0.30 to exclude any split not found in at least 30% of the bootstrap trees. Due to the inherently higher error rate of single molecule sequencing when compared to simultaneous sequencing of local clusters of the same molecule (Dohm et al. 2020), we sought to benchmark the use of our non-amplified ONT-generated gene sequences used for the phylogenies. We did this by Sanger sequencing of the LSU and SSU genes, using these as assumed ‘correct’ representations of the true DNA sequence.

We assumed that the read accuracy for the R9.4 flow cell used here should be around 90% (Wick et al. 2019). To evaluate which of our genes showed sufficient coverage supporting a tolerable error rate, we simulated batches of DNA reads with different final coverages with respect to the target sequence, using the LSU and SSU loci from *Purpureocilium atlanticum* sp. nov. as a reference. We employed NanoSim (Yang et al. 2017), which models the basecalling errors of ONT reads to inform the simulation of sequences with similar error characteristics. For the characterisation step, in which the model requires a training set, we used as input our trimmed, minimum Q9 reads that had been mapped to the respective genes in the magicBLAST step. We set the Sanger-sequenced gene as the reference.

To attain comparable coverage at the ends of our simulated reads (i.e., to support ONT reads that extended beyond our reference gene sequence), we required flanking sequences. Appropriate high-quality reads of sufficient similarity were determined by running BLAST searches of a few of the ONT reads that extended beyond the gene region, identifying *P. takamizusanense* strain PT3 chromosome 12 (Genbank CP086365.1) as the closest match. We used this reference to add 100bp of artificial high similarity flanking region to either end of the reference read. We then ran the characterisation step in ‘genome’ mode with default settings. For the simulation step, we used the error profiles output from the characterisation step in conjunction with the flanking region-enhanced reference genome, setting guppy-flipflop basecalling and minimum read length to the 95th quantile of the fragments’ respective read length distributions obtained in the experimental data. We reproduced this for different scenarios of sequence depth (2, 5, 10, 25, 50 and 75), replicating the experiment in triplicate.

We examined the output as consensus sequences in Geneious v2023.2.1 and summarised the difference in terms of polymorphisms observed between the Sanger reference and the consensus of the simulated reads to the Sanger reference gene. To compare the outcome with real measurements of depth in our reads mapped to fungal LSU and SSU genes, we mapped these to the respective Sanger sequences using minimap2 (Li 2018).

Supporting Figures


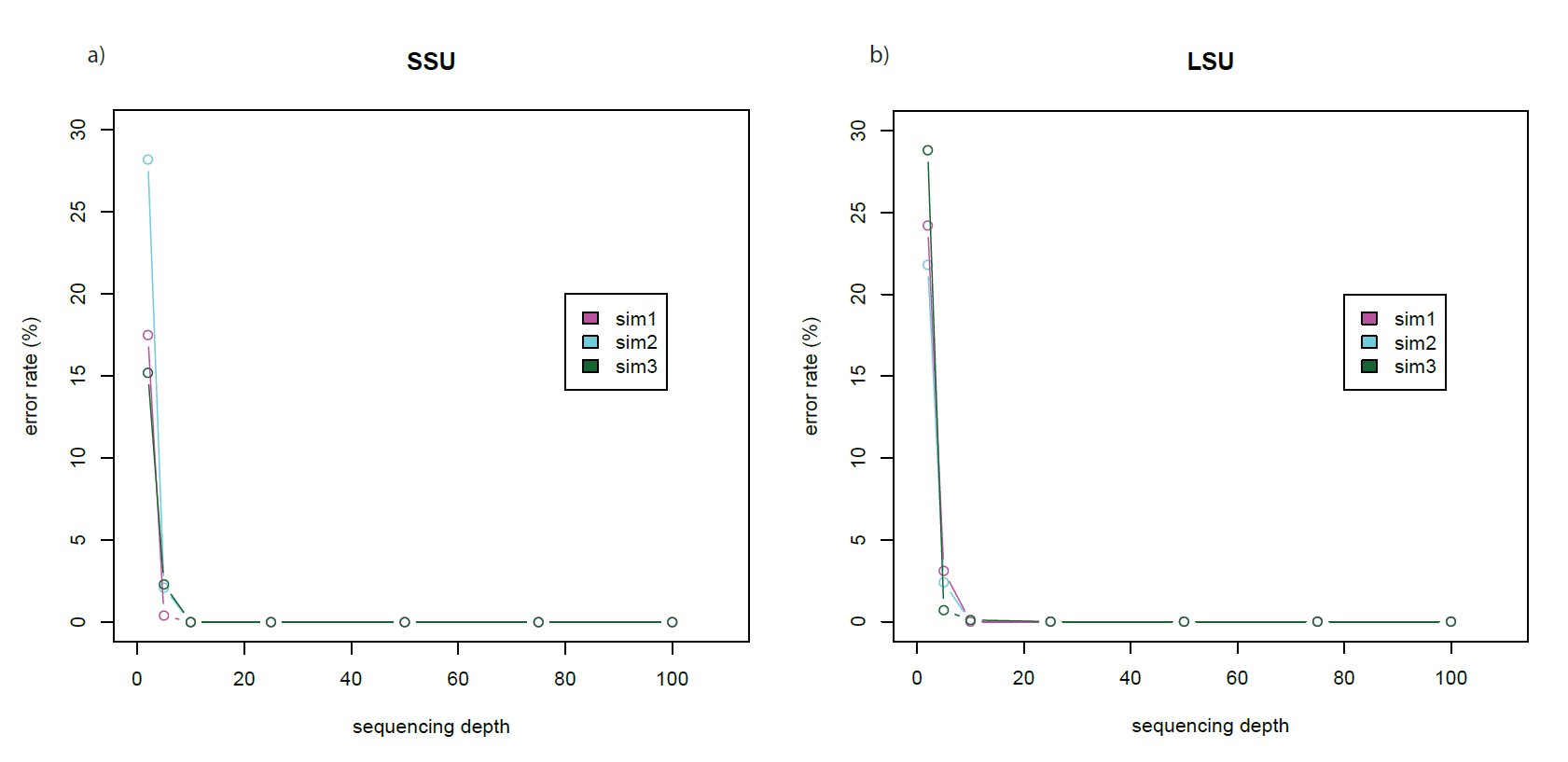


**Fig. S1.** Evaluating error rates for the fungal sequence data. Plots of error rate over full length of the SSU (a) and LSU (b) loci based on simulated Oxford Nanopore Technologies (ONT) reads covering these loci at depths 2, 5, 10, 25, 50 and 75, with three replicates of the simulation. Sanger-sequences of the SSU and LSU genes were used for benchmarking the rate of error in ONT reads.

**
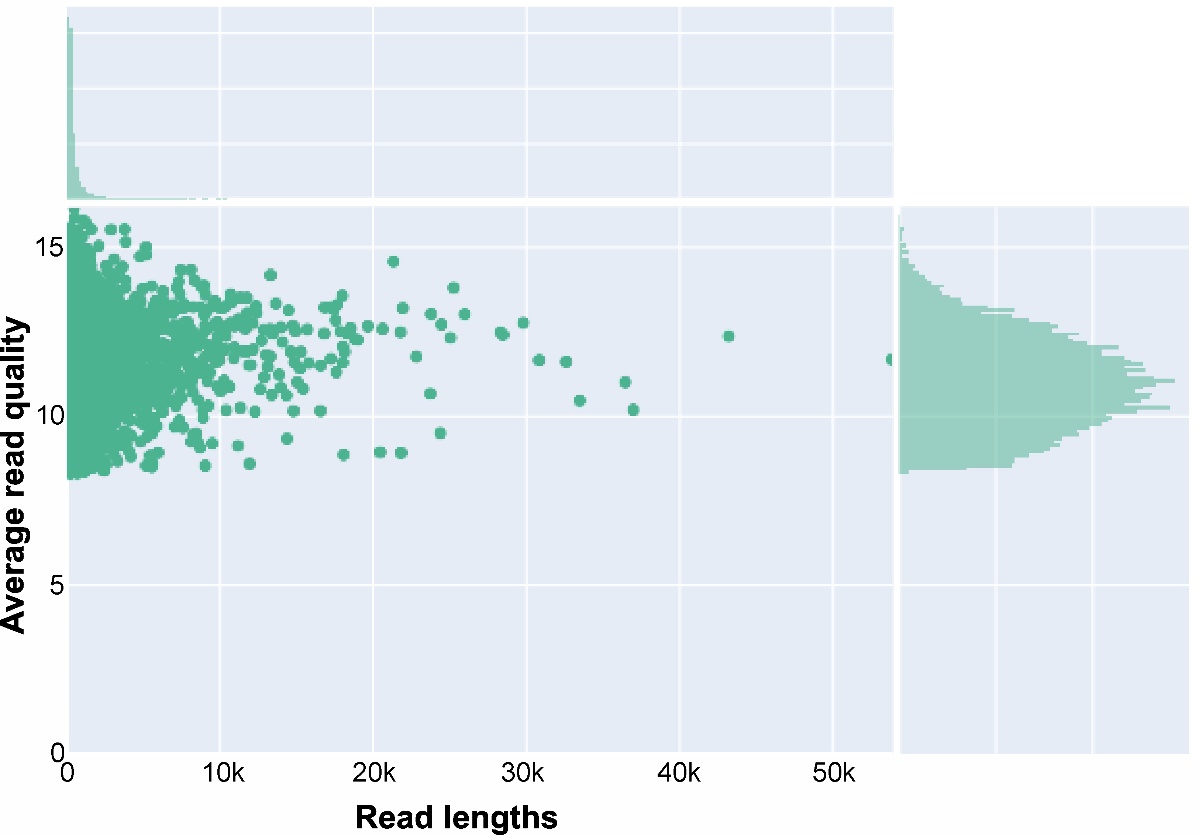
**

**Figure S2**. Read length vs average read quality plots for the sequence data produce from the fungi genomic libraries.

**
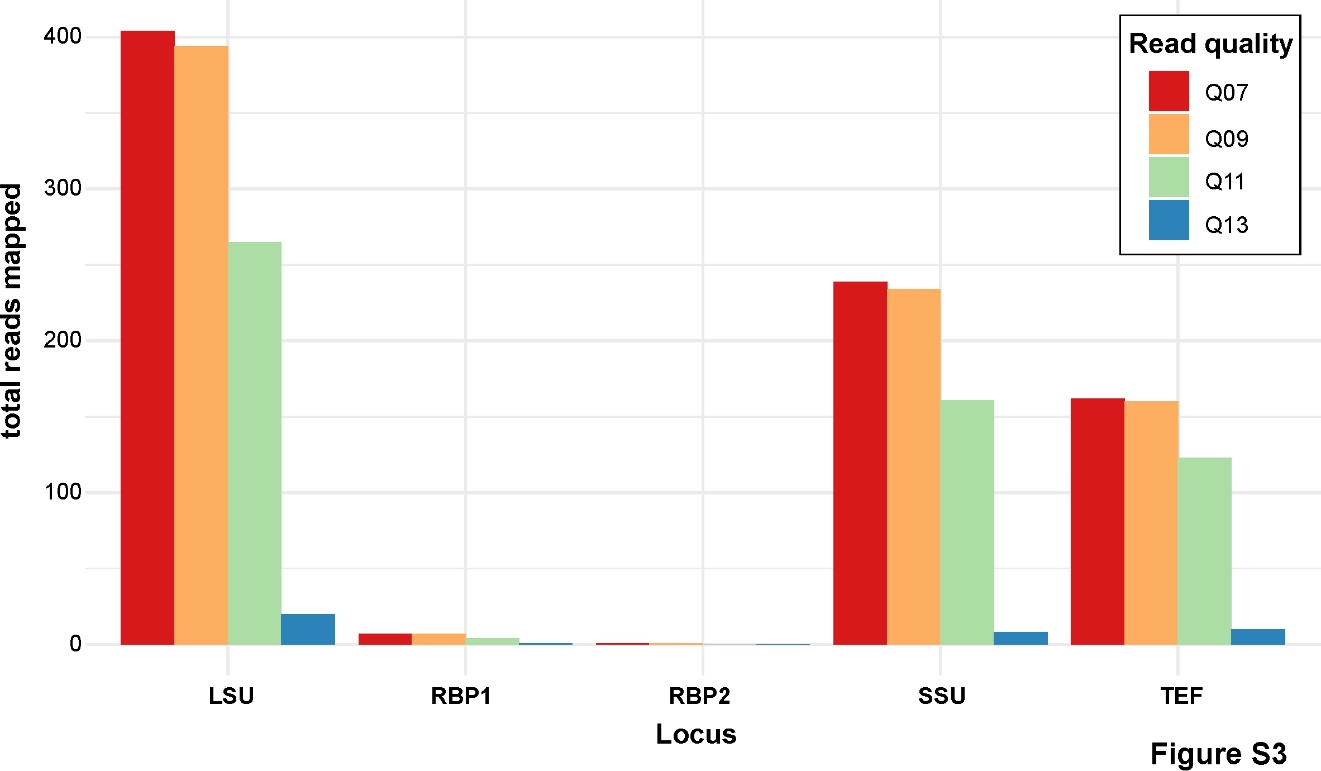
**

**Fig. S3.** Total reads mapped to ribosomal genes: large ribosomal subunit (LSU), RNA polymerase subunit gene 1, RNA polymerase subunit gene 2, small ribosomal subunit (SSU), translation elongation factor (*TEF*) in *P. atlanticum* sp. nov.

Supporting Tables

**Table S1.** DNA sequences retrieved from the NCBI repository of Hypocreales species and outgroups.

|  | **Fungi** |
| --- | --- |
| **Mean read length** | 870.8 |
| **Mean read quality** | 11 |
| **Median read length** | 278 |
| **Median read quality** | 10.9 |
| **Number of reads** | 240,000 |
| **Read length N50** | 3343 |
| **STDEV read length** | 2227.2 |
| **Total bases** | 208,999,028 |
| **Q5** | 240,000 (100%, 209 Mb) |
| **Q7** | 240,000 (100%, 209 Mb) |
| **Q10** | 177,333 (73%, 178.1 Mb) |
| **Q12** | 58,048 (24.2%, 83.7 Mb) |
| **Q15** | 614 (0.3%, 0.6 Mb) |

**Table S2.** Proportion of endogenous DNA retrieved for *P. atlanticum* sp. nov.

| Chromosome | Size (bp) | Number of genes recovered | Average bp recovered per gene | Coverage (bp) | Coverage (%) | Min coverage (bp) | Max coverage (bp) |
| --- | --- | --- | --- | --- | --- | --- | --- |
| CP086354.1 | 6,109,532 | 408 | 1,583.47 | 650,773 | 0.107 | 1 | 8 |
| CP086355.1 | 5,812,699 | 252 | 1,478.45 | 374,233 | 0.064 | 1 | 8 |
| CP086356.1 | 3,467,122 | 227 | 2,054.3 | 470,424 | 0.136 | 1 | 8 |
| CP086357.1 | 3,094,299 | 195 | 1,672.52 | 323,431 | 0.105 | 1 | 7 |
| CP086358.1 | 2,964,418 | 204 | 1,792.37 | 363,299 | 0.123 | 1 | 9 |
| CP086359.1 | 2,695,859 | 190 | 1,534.89 | 290,211 | 0.108 | 1 | 7 |
| CP086360.1 | 2,421,391 | 175 | 19,641.3 | 4,817,441 | 1.990 | 1 | 218 |
| CP086361.1 | 2,396,511 | 118 | 1,659.81 | 199,689 | 0.083 | 1 | 8 |
| CP086362.1 | 2,226,199 | 165 | 1,737.95 | 285,346 | 0.128 | 1 | 7 |
| CP086363.1 | 1,331,511 | 60 | 1,858.62 | 112,845 | 0.085 | 1 | 7 |
| CP086364.1 | 1,096,568 | 58 | 1,860.29 | 105,813 | 0.096 | 1 | 8 |
| CP086365.1 | 1,033,788 | 32 | 44,750.2 | 2,065,242 | 1.998 | 1 | 219 |
| CP086366.1 | 585,801 | 18 | 1,977.87 | 35,600 | 0.061 | 1 | 7 |
| CP086367.1 | 338,317 | 13 | 2,924 | 37,152 | 0.110 | 1 | 6 |
| **Total coverage** | **35,574,015** | **2,115** | **8,113,233** | **10,131,499** | **0.285** |  |  |

**Table S3.** DNA sequences retrieved from the NCBI repository of Hypocreales species and outgroups. Due the large size of this table, we have made it available, upon acceptance, at <https://figshare.com/s/17e1381832ba618b9051>.

References

Andrews S. 2010. Fastqc: A quality control tool for high throughput sequence data. Available from: http://www.bioinformatics.babraham.ac.uk/projects/fastqc

Araújo JPM, Evans HC, Kepler R, Hughes DP. 2018. Zombie-ant fungi across continents: 15 new species and new combinations within *Ophiocordyceps*. I. Myrmecophilous hirsutelloid species. Stud Mycol. 90:119-160.

Araújo JPM, Lebert BM, Vermeulen S, Brachmann A, Ohm RA, Evans HC, Debekker C. 2022. Masters of the manipulator: Two new hypocrealean genera, *Niveomyces* (Cordycipitaceae) and *Torrubiellomyces* (Ophiocordycipitaceae), parasitic on the zombie ant fungus *Ophiocordyceps camponoti*-*floridani*. Persoonia - Molecular Phylogeny and Evolution of Fungi. 49(1):171-194.

Boratyn GM, Thierry-Mieg J, Thierry-Mieg D, Busby B, Madden TL. 2019. Magic-blast, an accurate rna-seq aligner for long and short reads. BMC Bioinformatics. 20(1):405.

Camacho C, Coulouris G, Avagyan V, Ma N, Papadopoulos J, Bealer K, Madden TL. 2009. Blast+: Architecture and applications. BMC Bioinformatics. 10:1-9.

De Coster W, D’Hert S, Schultz DT, Cruts M, Van Broeckhoven C. 2018. Nanopack: Visualizing and processing long-read sequencing data. Bioinformatics. 34(15):2666-2669.

Dohm JC, Peters P, Stralis-Pavese N, Himmelbauer H. 2020. Benchmarking of long-read correction methods. NAR Genomics and Bioinformatics. 2(2).

Kans J. 2024. Entrez direct: E-utilities on the unix command line. Entrez Programming Utilities Help [Internet]. Bethesda (MD): National Center for Biotechnology Information (US).

Katoh K, Standley DM. 2013. Mafft multiple sequence alignment software version 7: Improvements in performance and usability. Mol Biol Evol. 30(4):772-780.

Li H. 2018. Minimap2: Pairwise alignment for nucleotide sequences. Bioinformatics. 34(18):3094-3100.

Li H, Handsaker B, Wysoker A, Fennell T, Ruan J, Homer N, Marth G, Abecasis G, Durbin R, Subgroup GPDP. 2009. The sequence alignment/map format and samtools. Bioinformatics. 25(16):2078-2079.

Stamatakis A. 2014. Raxml version 8: A tool for phylogenetic analysis and post-analysis of large phylogenies. Bioinformatics. 30(9):1312-1313.

Wick RR, Judd LM, Gorrie CL, Holt KE. 2017. Completing bacterial genome assemblies with multiplex minion sequencing. Microbial Genomics. 3(10).

Wick RR, Judd LM, Holt KE. 2019. Performance of neural network basecalling tools for oxford nanopore sequencing. Genome Biology. 20(1):129.

Yang C, Chu J, Warren RL, Birol I. 2017. Nanosim: Nanopore sequence read simulator based on statistical characterization. GigaScience. 6(4).
